# Subversion of Phytomyxae Cell Communication With Surrounding Environment to Control Soilborne Diseases; A Case Study of Cytosolic Ca^2+^ Signal Disruption in Zoospores of *Spongospora subterranea*

**DOI:** 10.3389/fmicb.2022.754225

**Published:** 2022-03-01

**Authors:** Jonathan Amponsah, Robert S. Tegg, Tamilarasan Thangavel, Calum R. Wilson

**Affiliations:** ^1^Tasmanian Institute of Agriculture, University of Tasmania, Hobart, TAS, Australia; ^2^Biotechnology and Nuclear Agricultural Research Institute Centre, Ghana Atomic Energy Commission, Accra, Ghana

**Keywords:** Ca^2+^ signaling, *Spongospora subterranea*, chemotaxis, motility, zoospore

## Abstract

Ca^2+^ signaling regulates physiological processes including chemotaxis in eukaryotes and prokaryotes. Its inhibition has formed the basis for control of human disease but remains largely unexplored for plant disease. This study investigated the role of Ca^2+^ signaling on motility and chemotaxis of *Spongospora subterranea* zoospores, responsible for root infections leading to potato root and tuber disease. Cytosolic Ca^2+^ flux inhibition with Ca^2+^ antagonists were found to alter zoospore swimming patterns and constrain zoospore chemotaxis, root attachment and zoosporangia infection. LaCl_3_ and GdCl_3_, both Ca^2+^ channel blockers, at concentrations ≥ 50 μM showed complete inhibition of zoospore chemotaxis, root attachment and zoosporangia root infection. The Ca^2+^ chelator EGTA, showed efficient chemotaxis inhibition but had relatively less effect on root attachment. Conversely the calmodulin antagonist trifluoperazine had lesser effect on zoospore chemotaxis but showed strong inhibition of zoospore root attachment. Amiloride hydrochloride had a significant inhibitory effect on chemotaxis, root attachment, and zoosporangia root infection with dose rates ≥ 150 μM. As expected, zoospore attachment was directly associated with root infection and zoosporangia development. These results highlight the fundamental role of Ca^2+^ signaling in zoospore chemotaxis and disease establishment. Their efficient interruption may provide durable and practical control of Phytomyxea soilborne diseases in the field.

## Introduction

Many Phytomyxean species are obligate endophytic biotrophs that parasitize a wide range of organisms ranging from flowering plants, algae, diatoms to several oomycetes in both marine and terrestrial environments (Neuhauser et al., 2011). They may be direct agents of diseases, notable among them are *Plasmodiophora brassicae* and *Spongospora subterranea* which causes clubroot in crucifers (Dixon, 2014), and powdery scab disease in potato (Falloon et al., 2015), respectively. Phytomyxeans are also known vectors of at least 20 plant viruses, including Oat mosaic virus (Hebert and Panizo, 1975), Sorghum chlorotic stunt virus (Kanyuka et al., 2003), Soil-borne wheat mosaic virus (Canova, 1996) and Potato mop top virus (Jones and Harrison, 1969). These phytomyxid and phytomyxid-vectored diseases are responsible for major economic losses in vegetable, oil seed and cereal crops globally (Kanyuka et al., 2003; Dixon, 2014; Wilson, 2016).

Phytomyxid infections are initiated by motile zoospores released from thick-walled, uninucleate resting spores of the sporogenic phase of a biphasic life cycle (Bulman and Neuhauser, 2016). Soon after release, the zoospores propelled by their heterokont flagella move in soil water or the marine environment to locate their hosts (Harrison et al., 1997; Bulman and Neuhauser, 2016). Zoospore movement can be directed by several environmental stimuli, but chemical cues, such as root exudation products, are the most commonly used by zoospores for host location (Zentmyer, 1960; Rai and Strobel, 1966). These chemical cues are, however, unable to transcend the zoospore cell membrane, because they are either too large or too charged (Walrant et al., 2017). Thus, just as in more complex organisms, these unicellular organisms have evolved elaborate mechanisms to perceive signals (Renaud et al., 2004), comprising the transduction triad of receptors, transducers, and effectors (Trewavas and Malho, 1997). Signals or primary stimuli are first perceived by transmembrane receptors, the transducers decode and relay the signal intracellularly to the effectors for appropriate action (Trewavas and Malho, 1997).

Many signal transduction pathways utilize secondary messengers, small, non-protein molecules, as transducers (Demaurex and Nunes, 2016). Evidence from eukaryotes and prokaryotes show that many physiological responses including chemotaxis, spore development, migration, virulence, and host pathogen interaction are mediated by secondary messenger molecules (Dominguez, 2004; Campbell et al., 2007; Asmat et al., 2014). Several molecules have been identified to have secondary messenger function in eukaryotic organisms (Pollard et al., 2017), with intracellular free calcium ions (Ca^2+^) the only molecule known to serve as a universal messenger in all eukaryotes (Campbell, 1983; Dominguez et al., 2015) and prokaryotes (Dominguez, 2004). In these organisms Ca^2+^ is ubiquitous in most transduction pathways and involved in a wide range of physiological and cellular functions (Demaurex and Nunes, 2016). In *Bacillus subtilis* for instance, the rotational movement of the flagella has been reported to be controlled by cytosolic Ca^2+^ (Szurmant and Ordal, 2004) while in *Escherichia coli* the role of transient Ca^2+^ on bacteria motility is well established (Tisa et al., 2000). Indirect evidence also supports the role of Ca^2+^ signaling on chemotaxis, with isoflavone attractants of *Phytophthora sojae* zoospores to soybean (Morris and Ward, 1992), found to stimulate the influx of Ca^2+^ into the zoospore cytosol from intracellular stores and the extracellular environment (Connolly et al., 1999).

Generally, intracellular influx of Ca^2+^ ions occur through various transport systems including antiporters and voltage-gated Ca^2+^-selective channels which have high conducting capacity, of approximately one million Ca^2+^ ions per second per channel (Clapham, 2007). This influx results in high cytosolic loading of Ca^2+^. Because Ca^2+^ overload will result in cell dysfunction and death (Carafoli and Krebs, 2016), eukaryotes and prokaryotes trigger extensive homeostatic systems to restore the intracellular Ca^2+^ load (Berridge et al., 2000) to remarkably low (*c.* 100 nM) concentrations at 20,000-fold less compared to the extracellular matrix by pumping Ca^2+^ into endoplasmic reticulum or out of the cell (Clapham, 2007). For every Ca^2+^ ion pumped out one ATP is hydrolyzed (Niggli et al., 1981). Dominguez (2004), in reviewing earlier studies that continuously monitored cytosolic Ca^2+^ concentration in *E. coli*, reported that in the presence of extracellular Ca^2+^ concentration within the mM range, intracellular levels steadily rose peaking at *c.* 2 μM before slowly declining to the initial level. This shows the fluidity of cytosolic Ca^2+^ concentration on signaling and lays the foundation for the exergonic principle underpinning Ca^2+^ flux.

Inhibition of Ca^2+^ flux across cellular systems have been found to constrain various physiological processes including chemotaxis and motility. Blocking Ca^2+^ channels of *E. coli* with ω-conotoxin GVIA, gallopamil or verapamil is reported to inhibit *E. coli* chemotaxis and motility (Tisa et al., 2000). Similarly, in *Spirochaeta aurantia*, chemotaxis was constrained by a Ca^2+^ channel inhibitor, botulinum toxin A (Goulbourne and Greenberg, 1983). Ca^2+^ also impacts the pattern of motility with the chelating Ca^2+^ ion in EGTA causing zoospores of *Pythium* spp. to swim in a straight line rather than the typical extended helical pattern interspersed with abrupt changes in direction (Donaldson and Deacon, 1993). Conversely, *in planta*, intracellular influx of Ca^2+^ has also been reported to constrain disease through the activation of defense responses such as rapid production of reactive oxygen species (Stael et al., 2015), phytoalexin production and accumulation (Mithöfer et al., 1999), as well as programmed cell death (Petrov et al., 2015). There are no known reports of the role of Ca^2+^ signaling in the physiological processes of *Phytomyxean* spp., but given the significance in other species, identifying the role of Ca^2+^ signaling on phytomyxid zoospore chemotaxis could provide a potential target for the development of a novel management strategy for their diseases. Thus, the study aimed at determining the role of Ca^2+^ signaling in the homing response of *S. subterranea* zoospores to host tissues. Therefore, in this study, using *S. subterranea* as an example of an important phytomyxid pathogen, we examined the effect of various Ca^2+^ antagonists on zoospore swimming patterns and correlated their impact on zoospore chemotaxis, root attachment and root infection.

## Materials and Methods

### *Spongospora subterranea* Inoculum Preparation, Incubation, and Zoospore Validation

Inoculum of *S. subterranea* was obtained from diseased tubers harvested from a commercial potato crop in Devonport, Tasmania, Australia (41.17°S, 146.33°E) in April 2018. Tubers were washed under running water to remove adhering soil and then air dried overnight at room temperature. The contents of individual lesions were scraped from the tuber surface with a scalpel blade minimizing removal of potato tissues, and the removed material oven-dried at 35^°^C for 7 days. Dried inocula was stored at room temperature for 2 months before use. To obtain zoospores, multiple 15 ml centrifuge tubes containing 100 mg of dried inoculum in 5 ml of deionized water were incubated for 3 months at room temperature. Any tubes found with excessive bacterial contamination were discarded. Zoospore identity was confirmed by microscopic examination of morphological features (spherical to ovoid cells measuring on average 4.77 ± 0.15 μM in diameter, biflagellate with bipolar short and long flagella) and typical helical swimming patterns of the zoospores in water (Merz, 1992, 1997; Balendres et al., 2016), and by qPCR testing (Thangavel et al., 2015). The swimming patterns were also observed and recorded at 59.94 fps using video-microscopy with Nikon D850 camera (Nikon Australia Pty Ltd., Auyuthaya, Thailand) fixed in position to the trinocular port of a Leica DMLB microscope (Lieca Microsystem, Wetzlar GmbH, Germany) in phase contrast mode (Supplementary Videos 1A–C). A computer-vision tracking software, idTracker, version 2.1 (Pérez-Escudero et al., 2014) was then used to analyze the recorded swimming video to ascertain zoospores spatial positions, XY, frame by frame and align them with their temporal positions, Z, for a 3D pattern analysis. This data was then used to construct a 3D trajectory plot using the data analysis software R version 4.0 (R Core Team, 2020) for a comparison with the zoospore swimming trajectory published by Merz (1992).

### Ca^2+^ Antagonists and Dose Setting

A range of compounds which interfere with Ca^2+^ cell signaling via different mechanisms were chosen and tested at various concentrations for their impact on *S. subterranea* zoospore motility. These compounds included lanthanum (III) chloride (LaCl_3_) and gadolinium (III) chloride (GdCl_3_), ethylene glycol-bis (β-aminoethyl ether)-N,N,N’,N’-tetraacetic acid (EGTA), trifluoperazine (TFP), and amiloride hydrochloride. Various concentrations of these compounds were tested for their effects on zoospore flagella movement. One microliter zoospore suspension was added to 5 μl of each test compound at 50–250 μM in a taxis chamber, mixed well and allowed 10 min to acclimatize before observing for zoospore flagella movement at 400 × using light microscope (Leica DMLB tilting trinocular compound microscope, Leica Microsystems, Wetzlar, Germany). This was repeated for TFP at 5–25 μM, and EGTA at 1,000–2,000 μM. Each trial was replicated five times. Chemical doses that caused a cessation of flagella movement were classified as lethal and excluded from subsequent trials.

### Ca^2+^ Antagonists Trial Doses

LaCl_3_ and GdCl_3_, both Ca^2+^ channel blockers (Katicheva et al., 2015), were tested at 50, 100, and 150 μM; EGTA, a Ca^2+^ chelator (Sharma et al., 1992), was tested at 100, 500, and 1,000 μM; amiloride hydrochloride, a Ca^2+^ flux inhibitor (Hedrich et al., 1988), was tested at 100, 150, and 200 μM; whilst TFP, a calmodulin antagonist (Vandonselaar et al., 1994), was tested at 2, 3.5, and 5 μM.

### Effect of Ca^2+^ Antagonists on Zoospore Chemotaxis

The Ca^2+^ antagonists were tested for their ability to interfere with *S. subterranea* zoospore chemotaxis using an adaptation of the traditional Adler capillary assay (Adler, 1973) by integrating cavitation into the solution within the microcapillary. In microcentrifuge tubes, 10 μl of a zoospore suspension (*c.* 11 zoospores/μl) was aliquoted, to which 50 μL of each Ca^2+^ antagonist at each tested concentration, or water only (control) was added. Tubes were gently vortexed and allowed to stand for 10 min. Glutamine (0.01 M), a known chemotaxis attractant for *S. subterranea* zoospores (Amponsah, 2021), was introduced into a 5 mm diameter microcapillary tube (Thermo Fisher Scientific, Loughborough, United Kingdom) with two open ends, such that the proximal tip contained approximately 10 μl of the glutamine solution (Supplementary Figure 1). Additional qualities of 0.01 M glutamine solution was then introduced into the distal end of the microcapillary tube using a 3 ml syringe in an intermittent manner to trap a series of air bubbles or voids within the column negating movement of the solution within the microcapillary by capillary action. A glutamine-filled cavitated microcapillary tube was then placed in each microcentrifuge tube containing the zoospore suspensions amended with various Ca^2+^ antagonists and incubated for 24 h in the dark at room temperature (20 ± 2°C). Each treatment was replicated six times. Following incubation, the tubes were removed from the zoospore suspension, the lower 10 μL of the solution within the microcapillary ejected onto a microscope slide and the total number of zoospores that had migrated to and entered each microcapillary tube was manually counted using light microscopy at 400 × magnification (Leica DMLB tilting trinocular compound microscope, Leica Microsystems, Wetzlar, Germany) scanning the entire sample.

Quantitative confocal live cell Ca^2+^ imaging and Microelectrode Ion Flux Measurement (MIFE) were also explored for the determination of the direct role of Ca^2+^ in *S. subterranea* zoospore motility and chemotaxis (details in Supplementary Experiments).

### Effect of Ca^2+^ Antagonists on Zoospore Root Attachment

Root tissues were excised from 6-week-old axenic tissue-cultured plantlets of potato cv. Iwa, a variety highly susceptible to *S. subterranea* root infection. Roots were triple rinsed in sterile deionized water, cut into *c.* 1 cm long pieces and placed two apiece onto a microscope slide. Each slide was then flooded with 45 μl of the various Ca^2+^ antagonist solutions followed by 15 μl of zoospore suspension (*c.* 11 zoospores/μl) added to the outer margins of the antagonist solution to prevent direct contact of the zoospore suspension with the root pieces and a cover slip carefully added. To prevent the drying, the prepared slide was placed on moistened Whatman filter paper (GE Healthcare, Chalfont Saint Giles, United Kingdom) within a Petri dish, to create high humidity in the chamber. Each Petri dish constituted a replicate with each treatment replicated six times. The set up was incubated for 12 h in a dark cabinet at room temperature (20 ± 2°C). Following incubation, the root pieces were carefully removed with forceps, gently rinsed in sterile deionized water to remove unattached zoospores, mounted on a microscope slide, and observed by light microscopy at 400 × magnification (Leica DMLB tilting trinocular compound microscope, Leica Microsystems, Wetzlar, Germany). The number of zoospores attached to the root and root hairs were counted.

### Impact of Ca^2+^ Antagonists on Root Infection

Solutions of each Ca^2+^ antagonist at each concentration were prepared using Hoagland’s nutrient solution, important to support plant hydroponic growth and a known stimulant of *S. subterranea* zoospore germination as a diluent (Falloon et al., 2003; Balendres et al., 2018). Ten ml of each solution was added to a sterile McCartney bottle to which was added 20 mg of resting spore inoculum with an estimated density ≥ 5,000 sporosori/mg and 30 μl of a zoospore suspension (*c.* 8 zoospores/μl) ensuring a ready and continual supply of zoospores over the course of the experiment. Each bottle was covered with aluminum foil to exclude excessive light and a the roots of a 6-week-old potato tissue-culture plantlet (cv. Iwa) was inserted into each bottle. The bottles were incubated in a plant growth chamber (Steridium Pty Ltd., Brisbane, Australia) set at 18^°^C, 90% relative humidity, 10,800 Lux light intensity, with a 14/10 h photoperiod for 8 weeks. The solutions were topped-up with 4 ml of Hoagland’s nutrient solution at 4 weeks.

After 8-weeks incubation, the plants were removed, the roots excised, and triple washed in running water. Five to eight root pieces of approximately 3 cm in length were sub-sampled from each of the upper, mid, and lower root regions of each plant root with six plants per treatment examined. Half of the collected root pieces were placed on glass microscope slides, stained with 0.1% trypan blue in lactophenol for 15 min, de-stained with water and mounted in glycerol. The stained root segments were observed, by light microscopy at 200 and 400 × magnifications, and scored for presence of *S. subterranea* zoosporangia within root epidermal cells and root hairs using a 0–5 rating scale (Hernandez Maldonado et al., 2013).

### Effects of Ca^2+^ Antagonists on Zoospore Swimming Patterns

Deionized water (control), Ca^2+^ antagonists at the concentrations previously used were individually added (15 μl) to a microcentrifuge tube to which 3 μl of zoospore suspension (*c.* 11 zoospores/μl) was added, the solutions then vortexed for 5 s and allowed to rest for a further 10 min. After this period, 3 μl of each treated zoospore suspension was pipetted into a taxis chamber (created by placing four cut glass cover slips onto a glass slide to create a chamber *c.* 0.18 mm deep over an area *c.* 1 cm^2^ to allow enough room for zoospore motility). The chamber was covered with a cover slip and observed under light microscope (Leica DMLB tilting trinocular compound microscope, Leica Microsystems, Wetzlar, Germany) at 400 × magnification. An individual zoospore was brought into focus and native videos of the swimming behavior of the zoospores were recorded at 59.94 fps via video-microscopy using a Nikon D850 camera (Nikon Australia Pty Ltd.) fixed in position to the trinocular port of the microscope set in phase contrast mode as described by Amponsah (2021). For each treatment, three replicated videos of different zoospores were recorded for analysis (Supplementary Videos 1A–I).

The native video files were standardized to 30 s whilst the quality was digitally optimized by cropping and sharpening images with Adobe Photoshop CC 2019 (Adobe, United States) to enhance image contrast to enable the tracking software to accurately follow zoospore movements. The recorded swimming behavior of the zoospores were tracked using a computer-vision tracking software, idTracker, version 2.1 (de Polavieja lab, Cajal Institute, Consejo Superior de Investigaciones Científicas, Madrid, Spain). The zoospores’ spatial positions, XY, were captured frame by frame and aligned with their temporal positions, Z, indicated by the frame number, for a 3D pattern analysis. A 3D trajectory plot from the data was reconstructed using the data analysis software Origin (Pro), Version 2018 (OriginLab Corporation, Northampton, MA, United States).

2D analysis of the same videos were done using the computer-vision tracking application, ToxTrac, version 2.84 (Rodriguez et al., 2018) with automated image-based tracking capacity. Zoospore quantitative swimming behavior in the optimized videos were tracked and analyzed to generate statistics on individual zoospore quantitative swimming parameters *viz* instantaneous speed (speed, μm/s), instantaneous acceleration (acceleration, μm/s^2^), motility rate, exploration rate [(Number of Areas)/(Number of Explored Areas (from Exploration)], and total distance traveled (distance, μm). 3D and 2D trajectory for each video were examined and compared among treatments to determine the impact on zoospore swimming pattern.

### Data Analysis

All were single factor experiments with treatments arranged in a completely randomized design. Data sets were tested for normality and the mean effects of replicated treatments were analyzed using a one-way ANOVA, with GenStat (12th Edition) or R statistical language framework v 4.0 (R Core Team, 2020). Where the *P*-value showed difference at 5% significance level, mean separation using LSD was done for all experiments except the root infection experiment where differences in infection intensity between treatments were tested using ordinal linear regression. This was done using R statistical language framework v 4.0 (R Core Team, 2020). A *post hoc* comparison was conducted using emmeans v1.2.3 (Lenth, 2018) and Tukey correction for pairwise comparison at 5% confidence level.

## Results

### Ca^2+^ Antagonists Dose Effects on Cessation of Zoospore Flagella Movement

Ca^2+^ antagonists treatments affected zoospore flagella movement in a dose dependent manner. The concentration that caused a cessation of active flagella movement varied among the compounds. In TFP zoospore flagella remained motile at 5 μM, but this movement ceased at concentrations ≥ 10 μM. Similarly, in amiloride hydrochloride zoospore flagella remained motile at 200 μM but ceased movement in concentrations ≥ 250 μM. In both LaCl_3_ and GaCl_3_ flagella motility was sustained at ≤ 150 μM but not at concentrations ≥ 200 μM. In EGTA flagella motility ceased only when concentration was increased to ≥ 1,200 μM.

### Effect of Ca^2+^ Antagonists on Zoospore Chemotaxis

**FIGURE 1:**
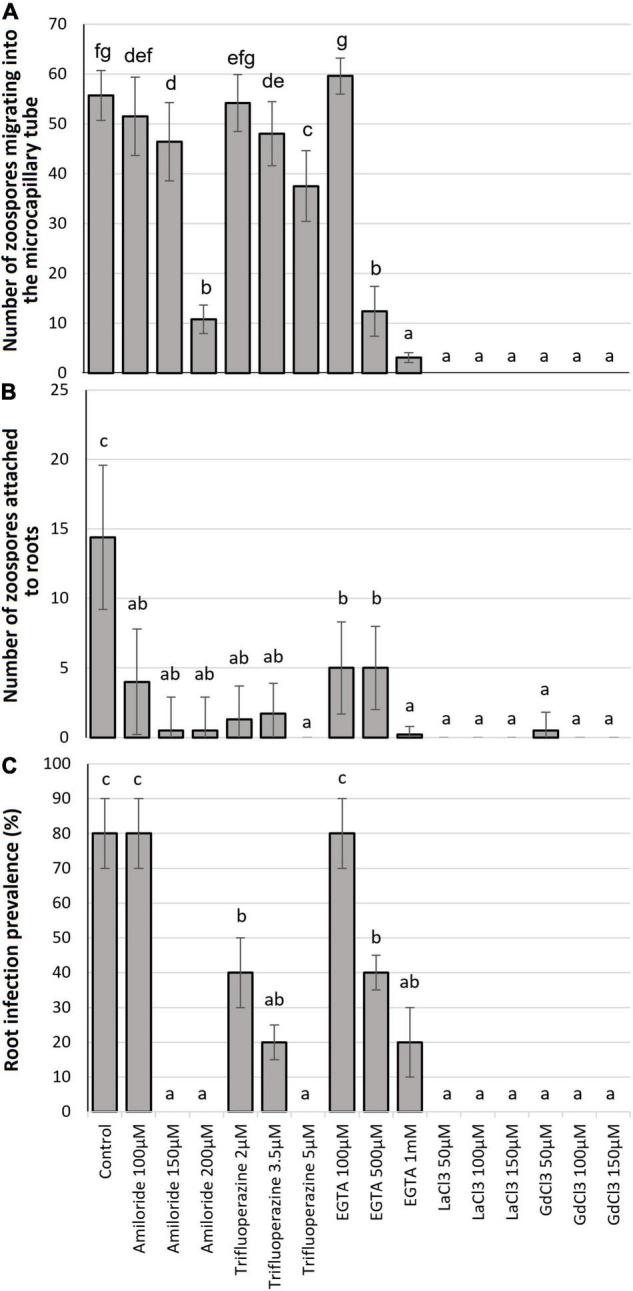
Effect of Ca^2+^ antagonist treatments: **(A)** chemotaxic attraction of zoospores to 0.01 M glutamine in a microcapillary assay; **(B)** zoospore attachment to potato roots after 24-h incubation; and **(C)** root infection prevalence 8 weeks after challenge. Bars headed with the same lowercase letter are not statistically different.

The number of zoospores that migrated into the microcapillary tube containing 0.01 M glutamine was significantly (*P* ≤ 0.05) diminished by all the Ca^2+^ antagonists at most of the tested concentrations when compared to the control (Figure 1A). The Ca^2+^ flux inhibitor, Amiloride hydrochloride significantly reduced chemotaxis at 150 μM (16.0%) and 200 μM (80.7%) concentrations but not at 100 μM (Figure 1A). The calmodulin antagonist TFP significantly reduced taxis at 3.5 μM (14.2%) and 5 μM (33.1%) concentrations but not at 2 μM, while the Ca^2+^ chelator EGTA significantly reduced chemotaxis at 500 μM (78.0%) and 1 mM (94.9%) concentrations but not at 100 μM. Migration of zoospores into the microcapillary tube was significantly inhibited by all concentrations of the Ca^2+^ channel blockers LaCl_3_ and GdCl_3_, all showing complete inhibition except 50 μM LaCl_3_ (Figure 1A).

### Effect of Ca^2+^ Antagonists on Zoospore Root Attachment

**FIGURE 2:**
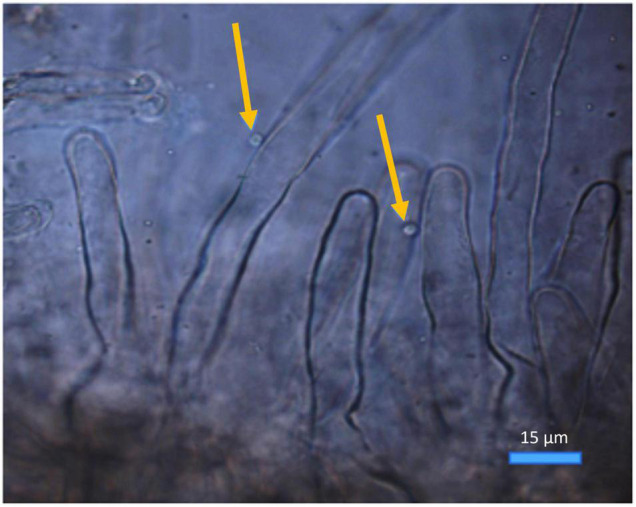
*Spongospora subterranea* zoospores (arrowed) attached to potato (cv. Iwa) root hairs following 24-h incubation (scale bar = 15 μm; magnification 400x).

The mean number of zoospores found attached to root segments (Figure 2) was significantly diminished (*P* ≤ 0.05) by all Ca^2+^ antagonist treatments at all concentrations when compared to the control (Figure 1B). Amiloride hydrochloride reduced root attachment by 75.2, 96.5, and 98.2% at 100, 150, and 200 μM concentrations, respectively, TFP by 90.3, 88.5, and 100% at 2, 3.5, and 5 μM concentrations, respectively, EGTA by 69.9, 69.0, and 98.2% at 100, 500, and 1 mM concentrations, respectively, and the 50 μM concentration of GdCl_3_ showing 96.5% reduction. Root attachment was completely prevented by all concentrations of LaCl_3_ and the 100 and 150 μM concentrations of GdCl_3_ (Figure 1B). It was also noted that with GdCl_3_ (100 and 150 μM) and LaCl_3_ (all concentrations) treatments zoospore motility was not observed.

### Effect of Ca^2+^ Antagonists on Root Infection

**FIGURE 3:**
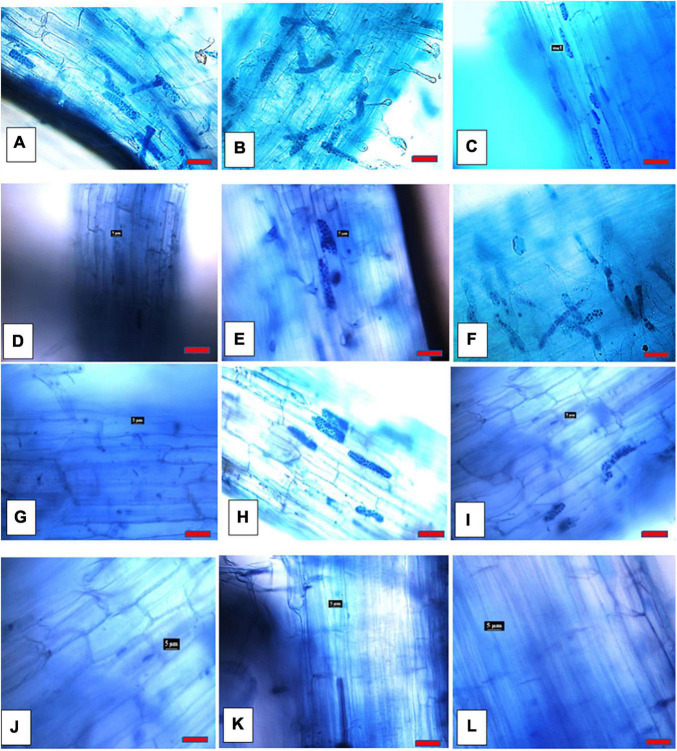
*Spongospora subterranea* zoosporangia root infection in potato (cv. Iwa) root 8 weeks following incubation with zoospore inoculum: **(A)** without Ca^2+^ antagonists; **(B)** 100 μM EGTA; **(C)** 500 μM EGTA; **(D)** 1 mM EGTA; **(E)** 100 μM amiloride hydrochloride; **(F)** 150 μM amiloride hydrochloride; **(G)** 200 amiloride hydrochloride; **(H)** 2 μM TPF; **(I)** 3.5 μM TPF; **(J)** 5 μM TFP; **(K)** 50 μM LaCl_3_; and **(L)** 50 μM GdCl_3_ (scale bar = 20 μm; magnification 400x).

There were no differences noted in infection levels from the three root regions analyzed (upper/mid/lower) and thus data was combined for each root. All Ca^2+^ antagonists significantly (*P* ≤ 0.05) diminished the prevalence (Figures 1C, 3) of zoosporangia root infection compared to the untreated control except for amiloride hydrochloride and EGTA both at 100 μM for which infection prevalence did not differ from the control (Figure 1C). TFP reduced disease prevalence by 60, 80, and 100% at 2, 3.5, and 5 μM concentrations, respectively, EGTA by 60 and 80% at 500 and 1 mM concentrations, respectively. Zoosporangia root infection was completely prevented by amiloride hydrochloride at both 150 and 200 μM concentrations and all concentrations of LaCl_3_ and GdCl_3_ (Figure 1C).

### Effect of Extracellular Ca^2+^ on Zoospore Chemotaxis

#### Effects of Ca^2+^ Antagonists on Zoospore Motility

All Ca^2+^ antagonists significantly affected the motility of *S. subterranea* zoospores in most measured parameters. Examples of native video files are provided in Supplementary Videos 1A–I. Zoospore swimming speed was significantly slower than the control for all treatments except for amiloride hydrochloride at 100 and 150 μM, EGTA at 100 and 500 μM and trifluoperazine at 2 and 3.5 μM which did not significantly differ from the control (Table 1). EGTA at 1,000 μM (9.16 μm/s), 5 μM trifluoperazine (7.58 μm/s) and 50 μM LaCl_3_ (2.1 μm/s) were significantly slower than the control. Zoospore motility completely ceased when treated with LaCl_3_ at 100 and 150 μM and GdCl_3_ at 50, 100, and 150 μM.

**TABLE 1:** Effect of Ca^2+^ inhibition treatments on zoospore swimming parameters.

	Swimming parameters				
Treatment	Speed (μm/s)	Acceleration (μm/s^2^)	Distance (μm)	Mobility rate	Exploration rate
Control	28.04 ± 1.30^cd^	31.88 ± 2.52^abc^	1002.5 ± 50.1^d^	0.9711 ± 0.025^a^	0.3333 ± 0.051^a^
Amil 100 μM	24.66 ± 1.58^cd^	57.21 ± 4.14^bcd^	742.1 ± 54.0^cd^	0.9965 ± 0.002^a^	0.3556 ± 0.044^a^
Amil 150 μM	19.25 ± 0.38^bcd^	35.4 ± 2.89^abc^	646.4 ± 54.0^bcd^	0.8437 ± 0.036^a^	0.2667 ± 0.019^a^
Amil 200 μM	9.44 ± 1.22^ab^	21.19 ± 1.66^ab^	288 ± 45.9^abc^	0.7404 ± 0.056^a^	0.1778 ± 0.029^a^
EGTA 100 μM	19.7 ± 1.16^bcd^	24.49 ± 0.39^ab^	591.9 ± 33.9^bcd^	0.9913 ± 0.009^a^	0.3333 ± 0.023^a^
EGTA 500 μM	15.96 ± 0.63^abc^	25.25 ± 2.32^ab^	479.3 ± 19.3^abc^	0.9989 ± 0.003^a^	0.3111 ± 0.040^a^
EGTA 1,000 μM	9.16 ± 1.21^ab^	14.48 ± 1.17^a^	275.4 ± 19.4^abc^	0.9661 ± 0.028^a^	0.1333 ± 0.019^a^
TFP 2 μM	33.23 ± 3.15^d^	92 ± 1.40^d^	1001.9 ± 94.2^d^	0.986 ± 0.011^a^	0.3333 ± 0.039^a^
TFP 3.5 μM	24.32 ± 1.17^cd^	65.6 ± 6.59^cd^	733.9 ± 34.5^cd^	0.9781 ± 0.022^a^	0.2000 ± 0.032^a^
TFP 5 μM	7.58 ± 1.48^ab^	14.48 ± 1.55^a^	229.5 ± 27.9^ab^	0.7248 ± 0.063^a^	0.1111 ± 0.022^a^
LaCl3 50 μM	2.1 ± 0.30^a^	14.13 ± 2.02^a^	75.2 ± 1.6^a^	0.5917 ± 0.041^a^	0.1111 ± 0.013^a^

*Zoospore swimming were digitally tracked for a quantitative determination of swimming parameters with an automated computer-vision tracking application (ToxTrac) after 10 min of exposure to Ca^2+^ antagonist treatments or water (control) for 30 s. Figures within the same column with the same superscripts are not statistically different (n = 3).*

Instantaneous acceleration (IA) of zoospores was not significantly affected by any treatment except trifluoperazine at 2 μM (92 μm/s^2^) which showed a significant increase in IA compared to the control (31. 88 μm/s^2^; Table 1). Ca^2+^ antagonists tended to reduce the total distance zoospores traveled compared to the control, but these differences were only significant for Amiloride hydrochloride at 200 μM, EGTA at 500 μM and 1 mM, TFP at 5 μM and LaCl_3_ at 50 μM (Table 1). Motility rate and exploration rate of zoospores treated with Ca^2+^ antagonists were not significantly different from the control (Table 1).

#### Effect of Ca^2+^ Inhibition on Zoospore Trajectory

**FIGURE 4:**
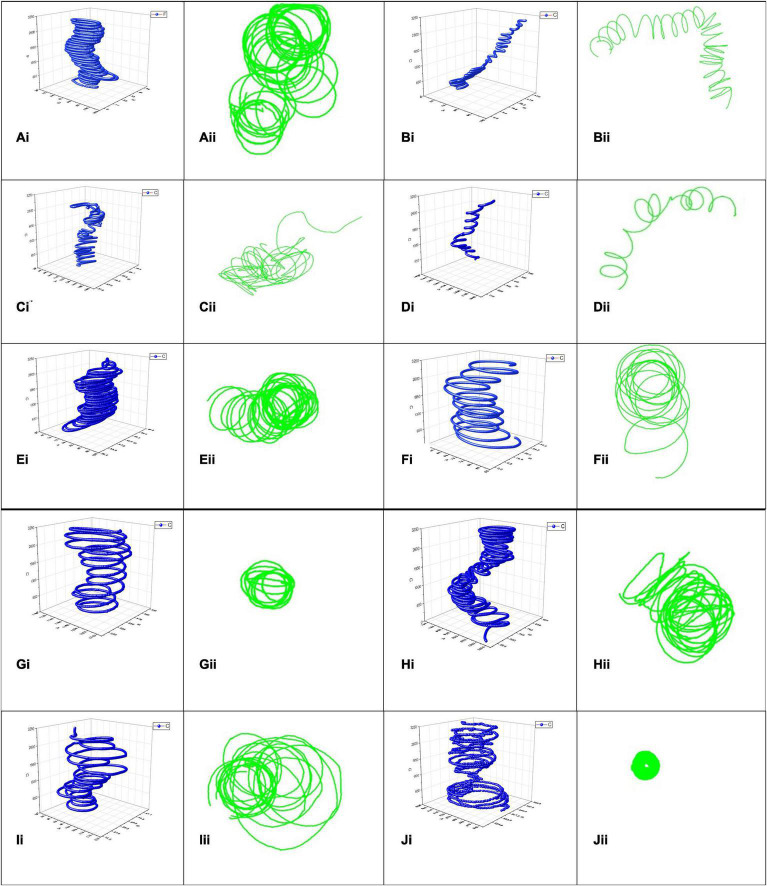
*Spongospora subterranea* zoospore swimming trajectories analyzed in 3D (blue) and 2D (green) when zoospores were treated with deionized water control **(Ai,Aii)**, 100 μM Amiloride **(Bi,Bii)**, 150 μM Amiloride **(Ci,Cii)**, 200 μM Amiloride **(Di,Dii)**, 100 μM EGTA **(Ei,Eii)**, 500 μM EGTA **(Fi,Fii)**, 1,000 μM EGTA **(Gi,Gii)**, 2 μM Trifluoperazine **(Hi,Hii)**, 3.5 μM Trifluoperazine **(Ii,Iii)**, and 5 μM Trifluoperazine **(Ji,Jii)**. Zoospore motility were tracked for 30 s with computer-vision tracking applications idTracker (blue) and ToxTrac (green), 10 min after acclimatizing in various Ca^2+^ antagonist treatments or water.

When subjected to the various Ca^2+^ antagonist treatments *S. subterranea* zoospores exhibited swimming patterns with moderate to substantial variation from the normal pattern demonstrated in the water control (Supplementary Videos 1A–C). The 3D mapped trajectory of zoospore movement in the control exhibited a characteristic helical swimming pattern (Figure 4Ai) with consecutive loops from the base of the vertical plane to the top. The 2D trajectory (Figure 4Aii) of the same video highlights four main whorls of rings in the horizontal plane, each whorl is constituted by a series of helical loops. The whorls represent the projected helical loop in the vertical plane of the 3D pattern. Conversely, zoospores treated with 100 μM Amiloride hydrochloride exhibited a combination of helical and oscillatory movement (Figures 4Bi,Bii). Movement typically begun with a few helical steps which changed to zig-zag oscillatory pattern and back to a helical pattern. This alternation of patterns became more apparent when zoospores switched from a vertical to horizontal planer movement. Increasing the concentration of amiloride led to greater disruption in swimming pattern. With 150 μM Amiloride treated zoospores, tracked trajectories showed a combination of oscillatory and meandering movement patterns (Figures 4Ci,Cii). More apparent in the 3D pattern, the zoospores movement at the base of the vertical plane was that of a zig-zag oscillatory pattern, midway through the plane the movement pattern changed to meandering movements. Motility was greatly curtailed when the concentration of Amiloride was increased to 200 μM. This was indicated by the reduced zoospore footprint as mapped out in the 3D trajectory (Figure 4Di). The pattern exhibited seemed to follow a pseudo-helical trajectory (Figure 4Dii) which differed from the other amiloride treatments as well as the control.

Like the controls, zoospores treated with 100 or 500 μM EGTA exhibited helical swimming patterns (Figures 4Ei,Eii,Fi,Fii) differing mainly in the density of the helical loops. Loops in the 500 μM EGTA treated zoospores (Figure 4Fi) were less dense compared to the 100 μM EGTA treated zoospores (Figure 4Ei). However, at 1,000 μM EGTA zoospore trajectory were more spiral than helical. The 3D trajectory (Figure 4Gi) of the zoospore movement showed the swimming pattern to be modeled on the shape of a truncated cone. A 2D trajectory of the same video indicated limited zoospore swimming movement in the horizontal plane (Figure 4Gii).

Generally, zoospore swimming trajectory became more convoluted when treated with trifluoperazine. In the 2 μM trifluoperazine treatment, the zoospore trajectory comprised of a combination of patterns (Figure 4Hi) including helical, oscillatory, and twisting patterns featuring a couple of intercalating loops. Rungs of successive loops were mostly close together, often with different orientations. The intensity of the convoluted trajectory was more apparent in the 2D trajectory of the same video (Figure 4Hii). At 3.5 μM trifluoperazine, however, zoospore trajectory was helical (Figure 4Ii), often differing in the sizes of the loop, adequately shown by the 2D trajectory of the same video (Figure 4Iii). Swimming pattern complexity reached its maximum when trifluoperazine concentration was increased to 5 μM (Supplementary Videos 1D–F). The swimming pattern followed a combination of trajectories including helical, meandering, twisting, and oscillatory patterns interspersed with paucity in movement giving rise to an overall outlook resembling an hourglass (Figure 4Ji). The 2D trajectory of the same video in the horizontal plane showed path resembling a hollow disc (Figure 4Jii). Zoospore movement ceased or were insufficient to map in treatments with LaCl_3_ and GdCl_3_ (Supplementary Videos 1G–I).

## Discussion

Many Ca^2+^ antagonists are known to constrain motility, chemotaxis, and other physiological processes in microbial organisms (Matsushita et al., 1988; Tisa et al., 2000; Islam and Tahara, 2001). These antagonisms have been exploited for medical treatment of several diseases including chlamydia (Ward and Salari, 1982) and neutrophils mediated atherosclerosis in humans (Shima et al., 2008). However, Ca^2+^ antagonism treatments are yet to be fully exploited for pathogenic plant disease control even though the role of Ca^2+^ signaling in various physiological processes of some plant pathogens are known. Here, we determined the role of Ca^2+^ signaling in *S. subterranea* zoospore chemotaxis, and proved Ca^2+^ antagonism to constrain motility, root attachment, and root infection. All four classes of Ca^2+^ antagonist tested (Ca^2+^ channel blockers, Ca^2+^ chelators, Ca^2+^ flux inhibitors and calmodulin antagonists) in this study were found to have antagonistic effects on zoospore chemotactic attraction to glutamine, root attachment and root infection in a dose dependent manner.

The cessation of chemotaxis of LaCl_3_ or GdCl_3_ treated zoospores at 50–150 μM concentration in this study is presumed to be due to their Ca^2+^ channel blocking activity. Other studies also found lanthanides such as La^3+^ and Gd^3+^ significantly constrain chemotaxis (Kinoshita et al., 2017; Wheeler, 2017). It was noted that by blocking Ca^2+^ channels in the plasma membrane and membranes of intracellular Ca^2+^ stores, the lanthanides limit the availability of free cytosolic Ca^2+^, the movement of which is required to phosphorylate ATP and drive motility and chemotaxis (Tisa and Adler, 1995). Conversely, the calmodulin proteins that bind Ca^2+^ and translate cytosolic Ca^2+^ transients associated with environmental stimuli also play crucial role in chemotactic response (Bothwell et al., 2006). Inhibiting calmodulin [“a multifunctional intermediate calcium-binding messenger protein” (Stevens, 1983)] function with trifluoperazine in this study reduced zoospore chemotaxis to glutamine along a concentration gradient. This agrees with Gauthier and O’Day (2001) who found a dose-dependent effect of trifluoperazine on *Dictyostelium* chemotaxis toward cAMP and folic acid.

The addition of EGTA generally reduced chemotaxis except at 100 μM which was not significantly different to the controls. It is conceivable that at 100 μM, EGTA was unable to chelate sufficient extracellular Ca^2+^ to impact on cytosol free Ca^2+^. In *Dictyostelium discoideum*, Schaloske et al. (2005) observed that 1 mM EGTA treatment was unable to produce perceptible change in the cytosol free Ca^2+^ concentration, however, 10 mM was enough to reduce the intracellular concentration significantly. Clapham (2007) argues that in low Ca^2+^ medium, plasma membrane Ca^2+^ ATPases pump Ca^2+^ out of the cell, to deplete the intracellular stores. It is this Ca^2+^ depletion that results in diminished chemotaxis. Inhibition of chemotaxis by amiloride hydrochloride such as we found with *S. subterranea* zoospores has been previously observed in the chemotactic response of neutrophils to N- formyl- methionyl- leucylphenylalanin (Simchowitz and Cragoe, 1986). Amiloride inhibits Ca^2+^ fluxes by blocking Na^+^-Ca^2+^ exchange (Teiwes and Toto, 2007) to limit cytosol free Ca^2+^.

Little is known of the physiological role of Ca^2+^ signaling in zoospore attachment, although recent evidence points to the regulatory role of Ca^2+^ in cell adhesion of pathogenic bacteria (Van Nhieu et al., 2018). The inhibition of cell-cell adhesion of human fibroblast with LaCl_3_ (Ko et al., 2001) provided the first direct evidence of the role of Ca^2+^ signaling on adhesion. At the organism level, adhesion and encystment of the unicellular protist Phytophthora zoospore is reported to be mediated by the secretion of proteins (Zhang et al., 2013). The relationship between Ca^2+^ signaling and adhesive protein secretion remains unknown. However, based on the dose dependent response of Ca^2+^ antagonist treatment on *S. subterranea* zoospore root attachment, it is conceivable to presume a Ca^2+^ signal interference in the adhesive secretion cascade. This may have led to interference with chemotaxis and root attachment. The prevalence of zoosporangia root infection is directly associated with the level of zoospore attachment, and thus the similarity of response of antagonists to these pathogen-host interactions is expected. Similar observation was made by Adorada et al. (2000) whilst studying the attachment of *P. capsici* zoospores to wounded pepper roots.

Given delimiting cytosolic Ca^2+^ concentration with a Ca^2+^ antagonist led to impairment of swimming behavior and chemotaxis, we can assume that for *S. subterranea* zoospores, swimming behavior is critical to chemotaxis and the regulation of Ca^2+^ concentration is central to it. However, the optimum Ca^2+^ concentration for swimming and chemotaxis is yet to be determined.

Directly linked to basic quantitative swimming behavior, is swimming trajectory. We found the normal extended helical movement punctuated with changes in direction (Merz, 1992) was affected differently by each Ca^2+^ antagonist. These changes in qualitative swimming behavior mirrored the changes in quantifiable behaviors. Ca^2+^ flux inhibitor or amiloride treatments changed the *S. subterranea* zoospore swimming pattern into more oscillatory movement which alternated frequently with helical and zig-zag patterns. In contrast, with *Phytophthora aphanidermatum*, Donaldson and Deacon (1993) reported that amiloride caused an irregular swimming pattern with repeated directional change. On the other hand, the Ca^2+^ chelator EGTA, did not alter the helical swimming patterns observed in the control, rather it reduced the density of the loops and thus diminished the distance traveled.

With the calmodulin antagonist, trifluoperazine, zoospore movement pattern appeared more convoluted, combining multiple swimming patterns perhaps indicating a state of confusion which culminated in frequent changes of direction at 2 μM. As the concentration increased the level of agitation reduced along with slowing down in speed. The characteristic alternation of acceleration from high to low and back to high, gave a distinct hourglass swimming pattern at 5 μM. Trifluoperazine also induced slow and spiral movements in *P. aphanidermatum* (Donaldson and Deacon, 1993), but in zoospores of *Achlya* spp., 5 μM trifluoperazine was sufficient to cause instant cessation of motility (Thomas and Butler, 1989). Various channel blockers have been known to produce motility effects similar to calmodulin antagonists (Tanida et al., 1986). The observed swimming pattern disruption described here may have originated from changes in flagella activity induced by limitations on concentration of cytosolic Ca^2+^ imposed by the various antagonist treatments. Since Ca^2+^ is the most important intracellular regulator for modulating flagella movement (Inaba, 2015) a limitation on its availability leads to disruption in flagella modulation (Smith, 2002). Though the mechanism underlying the differences in pattern induced by the different Ca^2+^ antagonist classes is beyond the scope of this study, the inherent differences in mechanism with which they achieve Ca^2+^ antagonism could be responsible for the differences in qualitative and quantitative swimming behaviors.

Overall, the further swimming pattern deviated from the helical trajectory observed in the controls, the greater the inhibition of chemotaxis, root attachment and zoosporangia root infection. This is consistent with observation in *P. aphanidermatum* where perturbation of the typical helical pattern into circular or straight-line movement with Ca^2+^ antagonists was associated with cessation of chemotaxis (Donaldson and Deacon, 1993).

## Conclusion

Chemotaxis of *S. subterranea* zoospores appear to be dependent on Ca^2+^ signaling. It is an important prerequisite for zoospore root attachment and zoosporangia root infection. Ca^2+^ channel blockers, chelators, flux inhibitors and calmodulin antagonists are all effective at inhibiting zoospore chemotaxis and reducing *S. subterranea* root attachment and root infection, but channel blockers, LaCl_3_ and GdCl_3_ at ≥ 50 μM provides the optimum inhibition. Ca^2+^ signaling affects both qualitative and quantitative swimming behaviors just as it affects chemotaxis, thus the rate of chemotaxis is proportional to the quantifiable zoospore swimming behaviors, notably speed, acceleration, and distance. Zoospores with helical swimming patterns, traveled longer distances at higher speed and acceleration and were the most chemotactically active. Detraction from the helical pattern to more oscillatory, twisted, or meandering patterns with the application of Ca^2+^ antagonists reduce all quantifiable swimming behaviors as well as chemotaxis. It remains, however, necessary to determine the effect of Ca^2+^ antagonists on the intracellular load of Ca^2+^ to confirm that Ca^2+^ antagonists operate by limiting the cytosolic concentration of Ca^2+^. The evidence above suggests Ca^2+^ antagonist as a potential treatment for limiting *S. subterranea* root infection.

## Data Availability Statement

The original contributions presented in the study are included in the article/Supplementary Material, further inquiries can be directed to the corresponding author/s.

## Author Contributions

JA, RT, TT, and CW designed the experiments. JA performed the experiments, analyzed the data, and prepared the original draft. CW, RT, and TT reviewed and edited the manuscript. CW was recipient of financial support for the project. All authors provided critical feedback to the article and approved the final version.

## Conflict of Interest

The authors declare that the research was conducted in the absence of any commercial or financial relationships that could be construed as a potential conflict of interest.

## Publisher’s Note

All claims expressed in this article are solely those of the authors and do not necessarily represent those of their affiliated organizations, or those of the publisher, the editors and the reviewers. Any product that may be evaluated in this article, or claim that may be made by its manufacturer, is not guaranteed or endorsed by the publisher.

## Supplementary Material

Supplementary Figure 1 Ca^2+^ antagonist anti-chemotaxis microcapillary assay set up.

Supplementary Videos 1 **(A–I)** Native videos of *S. subterranea* zoospores 10 min after suspension in deionized water control **(A–C)**, in 5 μM Trifluoperazine **(D–F)** or in 50 μM in LaCl_3_ **(G–I)**. Zoospore motility in **(A–C)** follows the typical helical pattern, those in **(D–F)** represents significant deviation from the typical pattern with reduce speed. **(G–I)** Shows oscillating movement pattern with no net propulsive movement.
