# Supplementary figures and images for: Subversion of Phytomyxae Cell Communication With Surrounding Environment to Control Soilborne Diseases; A Case Study of Cytosolic Ca2+ Signal Disruption in Zoospores of Spongospora subterranea
Source: Front Microbiol. 2022 Mar 1;13:754225. doi: 10.3389/fmicb.2022.754225 (PMC8921600; doi:10.3389/fmicb.2022.754225)

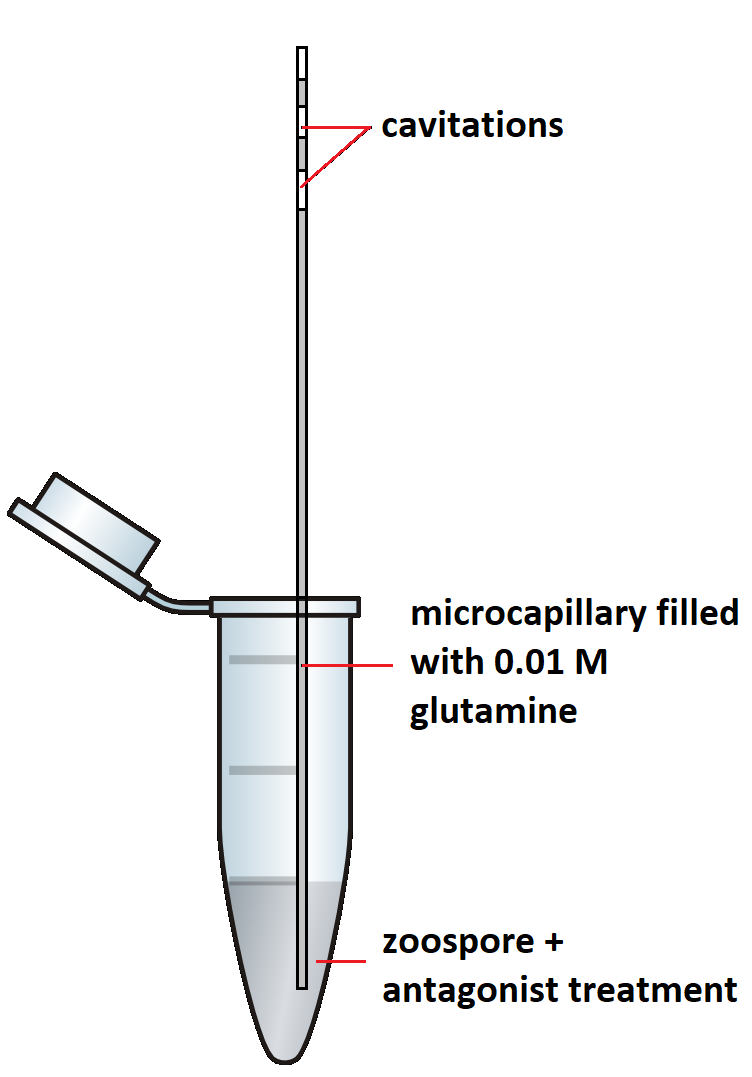

Supplement: Supplementary Figure 1 — Ca2+ antagonist anti-chemotaxis microcapillary assay set up. [file Image_1.TIF]
