# Supplementary material for: Subversion of Phytomyxae Cell Communication With Surrounding Environment to Control Soilborne Diseases; A Case Study of Cytosolic Ca2+ Signal Disruption in Zoospores of Spongospora subterranea
Source: Front Microbiol. 2022 Mar 1;13:754225. doi: 10.3389/fmicb.2022.754225 (PMC8921600; doi:10.3389/fmicb.2022.754225)
Supplement: Supplementary file 10 [file Data_Sheet_1.docx]

**Supplementary Experiments**

**Role of Ca^2+^ in Phytomyxea zoospore motility and chemotaxis**

1. **A confocal live cell Ca^2+^ imaging approach**

Quantitative confocal live cell Ca^2+^ imaging was explored for a determination of the direct role of Ca^2+^ in *S. subterranea* zoospore motility and chemotaxis. Here, zoospores previously suspended in deionised water or 0.01 M glutamine to activate motility were loaded with 5 μM Fura-2 acetoxymethylester (AM) or 5 μM Fluo-4- AM (Invitrogen, CA, USA) in Hanks balanced salt solution (HBSS) without phenol red (Sigma-Aldrich, MO, USA) for 45 mins or 2 hrs at 37^o^C. The zoospores were then washed in fresh HBSS followed by incubation in deionised water at 37^o^C for 2 hrs or overnight. Aliquots of the dye-loaded zoospore in deionised water were placed onto glass slides and covered with cover slips. Focusing on one zoospore at a time with an UltraView spinning disk confocal microscope equipped with UV and visible laser lines (351, 364 and 488 nm) with Volocity Software (Perkin Elmer, MA), confocal fluorescent images of the treated live zoospores were taken and processed for analysis.

1. **Microelectrode Ion Flux Measurement (MIFE) approach**

To measure the net Ca^2+^ fluxes at the cell membrane of *S. subterranea* during motility, released zoospores were immobilised on coverslips coated with poly-L-lysine according to methods by Shabala *et al*. (2002) with some modifications. Zoospore suspension (60 µl) of approximately 3 zoospore/ µl were aliquoted into 2 ml microfuge tubes and centrifuge at 130,000rpm for 10 minutes, followed by immediate decanting of the supernatant (~40 µl) in an attempt to increase the zoospore density. 10 µl of the concentrated zoospores was applied onto the coated coverslips and allowed ~3 minutes for the zoospores to settle. After, the coated slips with the immobilised zoospores were washed with running sterile deionised water to remove all unattached zoospores. The coverslips were then assembled with a taxis chamber and observed with light microscope at 400× magnification prior to Ca^2+^ flux measurements.

**Results and Discussion**

Florescence intensities of intracellular Ca^2+^ of both chemotactically stimulated and unstimulated zoospores were below the quantifiable limit irrespective of the fluorescent stain used or the duration of the incubation. The indicator dyes in the form of acetoxymethyl (AM) esters though required no invasive loading procedure however requires the lipid bilayer of the target cell/tissue/organism to be readily permeable to the dye (Russel, 2011). With limited knowledge on the *S. subterranea* zoospores cell membrane composition, it is unknown if the cell membrane was lipophilic enough to allow for sufficient passive diffusion of the dye, or the zoospores lacked sufficient intracellular esterase activity to cleave the dyes to make them active for high signal intensity. Thus, the reason underlying low signal strength is therefore open to speculation. Conversely, due to the labile nature of zoospores which makes them highly susceptible to environmental stresses, other dyes in other forms such as salts or dextran conjugate forms, both non-permeable to cell membrane could not be explored for confocal imaging due to their requirement of invasive loading procedures (e.g., patch clamp, microinjection electroporation etc.) which are likely to impact the zoospore viability during.

Zoospore immobilization on poly-L-lysine coated coverslip was achieved (Fig. 1), however the number of zoospores per unit area remained lower than the optimum density required for MIFE analysis despite the centrifugation step. Further density increment could not be attained as the obligate nature of the pathogen could not permit the pathogen to be cultured or multiplied outside the host. This notwithstanding, a reliable MIFE output requires a dense single layer of the target tissue or organism to reduce the background noise which impacts on the signal-to-noise ratio (Shabala *et al*., 2006). Without the required cell density MIFE could not be used to for the determination of the role of Ca^2+^ in *S. subterranea* zoospore motility and chemotaxis.


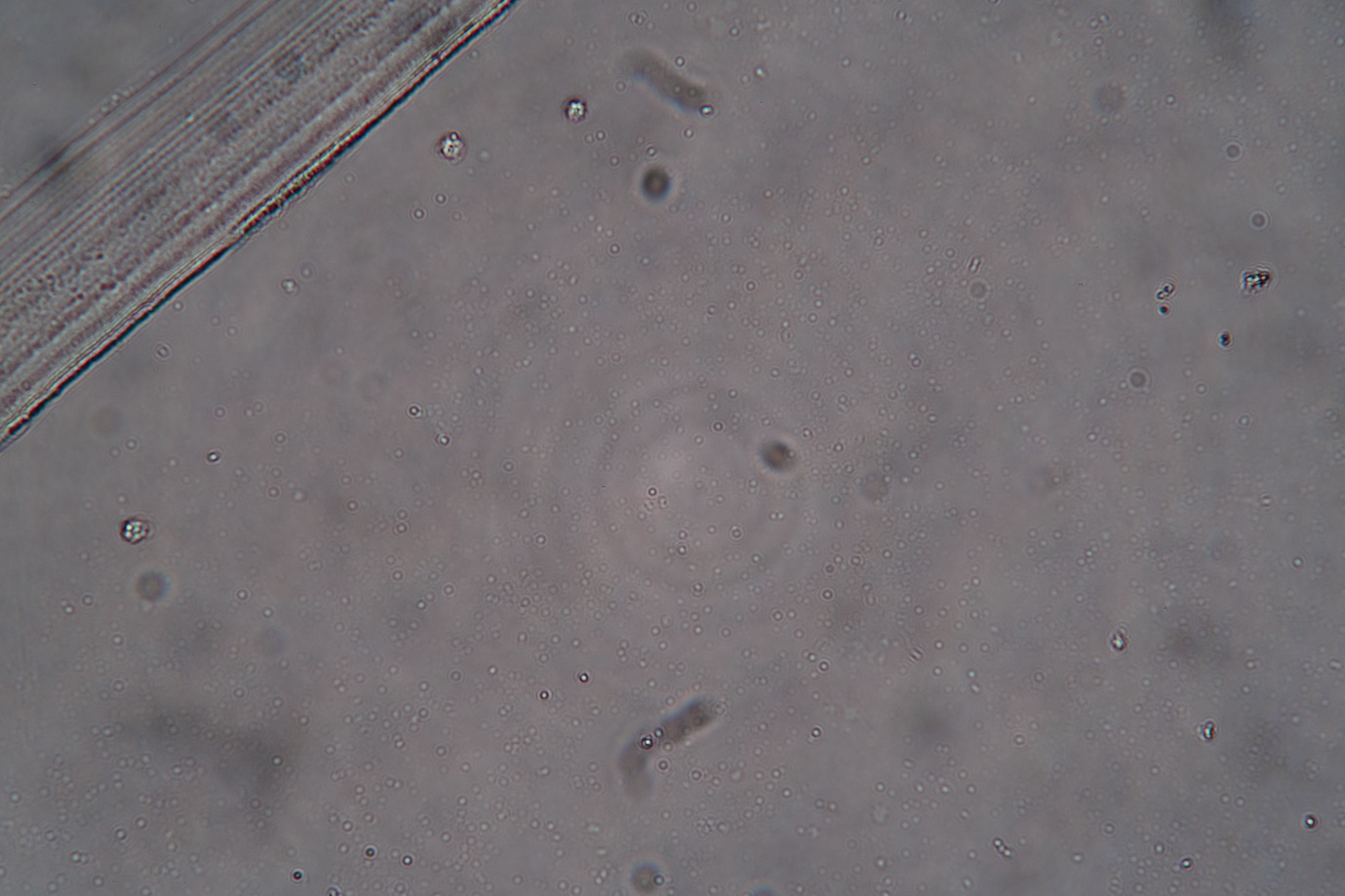


**Figure 1:** *S. subterranea zoospores* (arrowed) at 400× magnification immobilized on poly-L-lysine coated coverslip, scale bar = 16 µl.

**References**

Shabala, L., Ross, T., McMeekin, T., Shabala, S., (2006). Non-invasive microelectrode ion flux measurements to study adaptive responses of microorganisms to the environment. *FEMS Microbiology Reviews*, **30(3)**, pp.472-486.
